# A Mitochondrial Basis for Heart Failure Progression

**DOI:** 10.1007/s10557-024-07582-0

**Published:** 2024-06-15

**Authors:** William D. Watson, Per M. Arvidsson, Jack J. J. Miller, Andrew J. Lewis, Oliver J. Rider

**Affiliations:** 1 Division of Cardiovascular Medicine, University of Cambridge, Cambridge, UK; 2 Oxford Centre for Magnetic Resonance Research, University of Oxford, Oxford, UK; 3 Clinical Physiology, Department of Clinical Sciences Lund, Lund University, Lund, Sweden; 4 Department of Clinical Physiology, Skåne University Hospital, Lund, Sweden; 5 Department of Clinical Medicine, Aarhus University, Aarhus, Denmark

**Keywords:** Heart failure, Mitochondria, Redox, Calcium, ATP

## Abstract

In health, the human heart is able to match ATP supply and demand perfectly. It requires 6 kg of ATP per day to satisfy demands of external work (mechanical force generation) and internal work (ion movements and basal metabolism). The heart is able to link supply with demand via direct responses to ADP and AMP concentrations but calcium concentrations within myocytes play a key role, signalling both inotropy, chronotropy and matched increases in ATP production. Calcium/calmodulin-dependent protein kinase (CaMKII) is a key adapter to increased workload, facilitating a greater and more rapid calcium concentration change. In the failing heart, this is dysfunctional and ATP supply is impaired. This review aims to examine the mechanisms and pathologies that link increased energy demand to this disrupted situation. We examine the roles of calcium loading, oxidative stress, mitochondrial structural abnormalities and damage-associated molecular patterns.

## Introduction

In health, the heart has an incredible ability to respond directly to loading and demand changes to keep up with the body’s requirements. In addition, myocyte mitochondria are able to precisely match ATP supply with demand.

However, any heart placed under prolonged and excessive load will, over time, fail. This is used experimentally to induce heart failure (for instance, rapid pacing or aortic constriction) but is also seen clinically (high output heart failure, tachycardia-mediated cardiomyopathy or valvular heart disease leading to pressure or volume loading). This process involves mechanical remodelling of the ventricle, hypertrophy and fibrosis of the myocardium, inflammatory cell infiltrate and alterations to cellular pathways within myocytes themselves leading to hypertrophy and apoptosis. It affects all cells and components of the heart cardiomyocytes, fibroblasts, endothelium and interstitium [1, 2]. The geometry of the heart changes from an elliptical to spherical shape, further compromising its contractile function. After an initiating event, these mechanisms will cause constant progression of heart failure unless checked. The pathways involved are incompletely mapped and it is unclear which cause physiological adaptations to pressure or volume loading and which cause pathological adaptations [3, 4].

The aim of this review is to understand how hearts respond to load, the adaptive and maladaptive cellular responses that ensue in different high-load states and how mitochondria may play a role in the progression of heart failure.

## Energy Requirements of the Heart

### Mechanical Energy Requirements

From basic mechanical principles, we can ascertain that the work done by the left ventricle will be proportional to the volume of blood pumped and the pressure against which it must be pumped.

The most simplistic approach is rate pressure product (RPP), the heart rate multiplied by the systolic blood pressure.

**Fig. 1:**
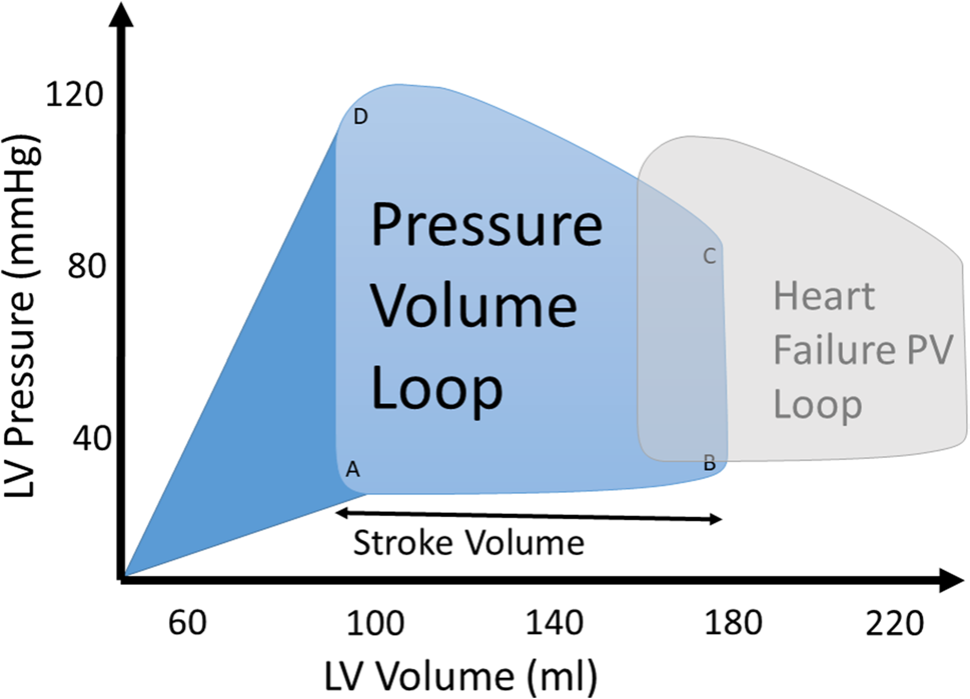
Pressure–volume loop and pressure–volume area. The PV loop begins at beginning of diasystole (**A**) and fills through diastole to point **B**. From B to C is the beginning of systole (isovolumic contraction) where pressure rises but the aortic valve is closed and volume does not change. At point **C**, left ventricular pressure exceeds aortic diastolic pressure and the aortic valve opens, leading to ejection and a fall in left ventricular volume down to point **D**, where the aortic valve closes. From D to A, left ventricular rapidly falls and the cycle begins again. The pressure–volume loop and the triangular dark shaded area to the left form the pressure–volume area (PVA). The smaller, grey loop to the right represents an example of the PV loop in heart failure

Plotting left ventricular pressure against volume for the cardiac cycle forms a loop (a pressure–volume loop) with its area corresponding to the mechanical work done in each cardiac cycle. By extending the loop to an intercept on the x-axis, this forms the pressure–volume area (PVA), which models the mechanical energy required within a stretching heart using a time-varying elastance model (Fig. 1). Suga’s group correlated PVA derived from pressure–volume loops recorded from isolated canine hearts with myocardial oxygen uptake (MVO_2_, an indicator of energy substrate oxidation and hence ATP demand) and found the correlation was good in an individual heart in the baseline state [5, 6] but varied from heart to heart [7] and with levels of catecholaminergic stimulation [8]. Total MVO2 will include the basal metabolism and energy expended in ion transport (predominantly calcium) which are independent of PVA, as well as the actin-myosin cross-bridge cycling which is proportional to PVA. Increased contractility is due to changes in calcium cycling hence shifts the relationship between MVO2 and mechanical work upwards [9].

The mechanical work done, which is quantified in PVA, is often referred to as ‘external work’ and the energy expended in basal metabolism and ion transport is referred to as ‘internal work’. The efficiency of the system may be defined as the ratio between ventricular stroke work and oxygen consumption [10].

In heart failure, the pressure–volume loop is shifted to the right owing to dilation of the ventricle, increasing PVA and hence energy requirements [11]. The relationship between mechanical dyssynchrony and oxygen consumption is more complicated: in acute studies, both oxygen consumption and mechanical work done increased following resynchronisation, however mechanical work increased more than oxygen consumption, hence making the ventricle more efficient [12], presumably down to global mechanical work not representing individual segmental work. Efficiency increased following aneurysmectomy [13].

Phosphorus-31 cardiac magnetic resonance subsequently allowed determination of ATP flux through the creatine kinase reaction in vivo, which has confirmed that ATP flux can vary significantly with loading conditions within the same heart in spite of minimal change in rate pressure product or PVA [14]. This suggests a large contribution to changes in ATP demand comes from alterations in internal work.

Modelling whole-heart energy requirement has proven challenging. Mechanical energy requirements may be similarly calculated for the right ventricle [15] and the atria [16] but how relationships change with geometric alterations to the LV is unclear. In addition, calculating the ATP requirements for internal work has yet to be done reliably. An overarching model to link myocardial energy consumption to the different factors affecting it is therefore lacking.

### How the Energy Demand Is Met

The process of transforming metabolic substrates into usable energy has been extensively reviewed elsewhere [17–19]. In brief: it can be divided into three stages of substrate metabolism, oxidative phosphorylation and phosphotransfer. Metabolic substrates are taken up by the heart, principally free fatty acids (60–90%) and glucose (10–40%) but with a lesser contribution from ketones, lactate and amino acids. Glucose enters glycolysis and fatty acids beta-oxidation to produce Acetyl Co-enzyme A which can enter the tricarboxylic acid (or Krebs) cycle. The tricarboxylic acid cycle produces the reducing intermediates NADPH and FADH2, which in turn feeds into the electron transport chain, creating an electron gradient across the inner mitochondrial membrane. This gradient is used by the enzyme F1F0 ATPase to produce ATP from ADP. To facilitate ATP transport around the cell, ATP transfers a phosphate group to creatine to form phosphocreatine, which can rapidly move to sites of ATP demand, where it reacts with ADP to re-form ATP.

Two abilities are key. First, keeping up a high rate of ATP production that matches the heart’s constantly changing demand. The second, related ability is to retain a large concentration difference between [ATP] and [ADP]. This determines the energy of hydrolysis of ATP (ΔG_ATP_): enzymes require ΔG_ATP_ to remain above a certain amount to function, for instance while actin-myosin cross-bridge cycling requires a large turnover of ATP, SERCA requires a higher ΔG_ATP._ These factors are determined by both mitochondrial activity and a system of phosphotransfer, which shuttles high-energy phosphate to its sites of use.

## How Energy Supply and Demand Are Matched

The heart has a remarkable ability to match ATP synthesis to hydrolysis with cardiac oxygen consumption, increasing proportionately with workload but intracellular ATP concentrations and ΔG_ATP_ remaining constant [20]. In the healthy heart, PCr/ATP remains constant under moderate stress, [21] [22] suggesting that at these workloads there is sufficient ability to increase substrate metabolism, oxidative phosphorylation and phosphotransfer rates to meet the increased ATP demand. Multiple processes (calcium levels, AMP signalling, coupled reactions) interact to produce this balance [23].

### High-Energy Phosphate Metabolism and ATP

**Fig. 2:**
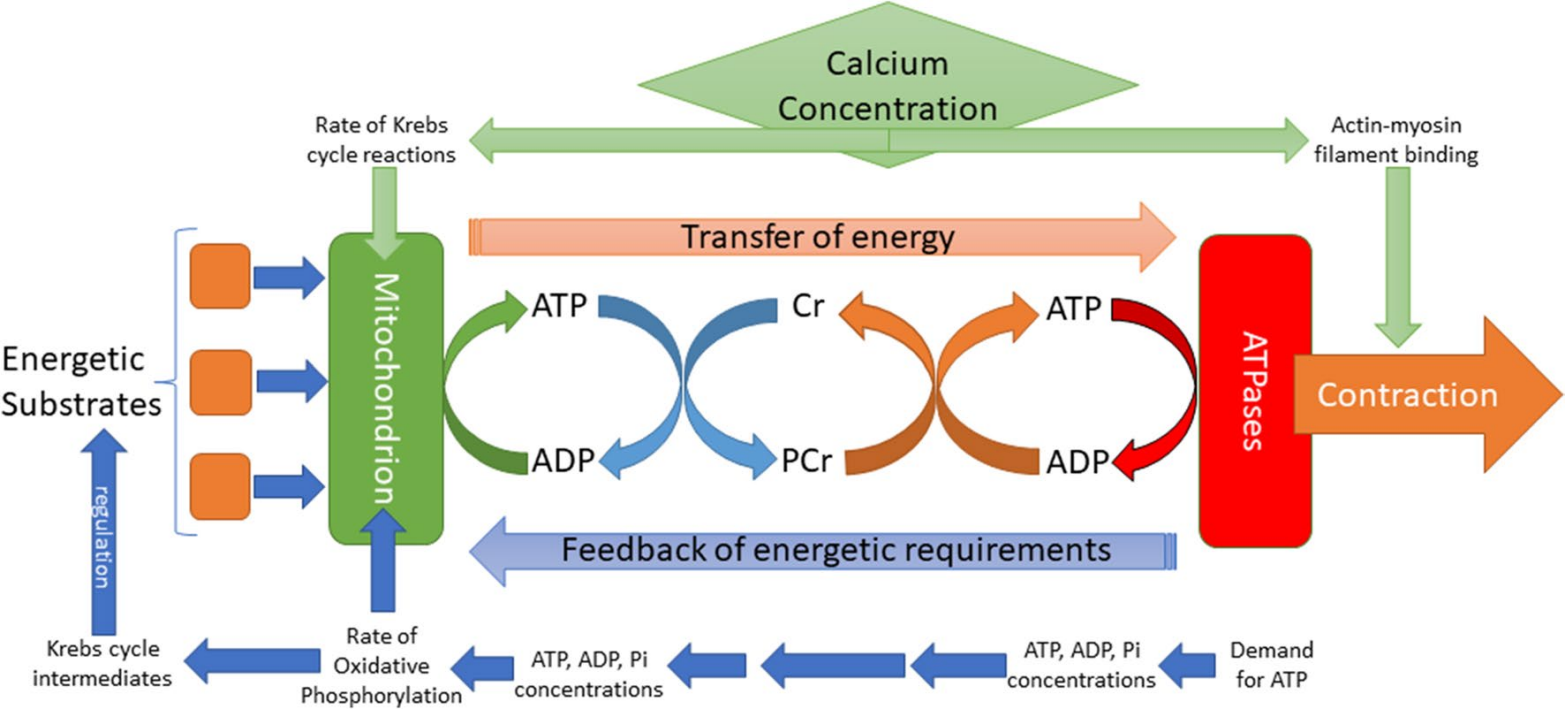
Diagrammatic illustration of the linkage between ATP supply and demand via phosphate shuttles and with regulation by calcium. Changes in concentrations of ATP, ADP and phosphate (Pi) will shift the equilibrium of the creatine kinase reaction, increasing ADP near mitochondria and stimulating oxidative phosphorylation and the Krebs cycle. Increases in intracellular calcium stimulate both contraction and mitochondrial ATP production, harmonising the two processes

The role of phosphorus in energy transfer is summarised in Fig. 2. Increased ATPase activity alters ATP/ADP balance at sites of use, with the phosphotransfer mechanism also acting as a feedback system to change this balance within the mitochondria or other sensitive areas. An increase in reaction substrates at the mitochondrion (ADP and Pi) increases the rate of oxidative phosphorylation [24, 25], which in turn leads to a change in flux through the Krebs cycle and a decrease in Krebs cycle intermediates and other reactants. For instance, a fall in Acetyl CoA leads to an increase in fatty acid transport, while a fall in citrate stimulates glycolysis [23, 26].

AMPK (AMP-activated protein kinase) promotes ATP production during higher workloads or oxidative stress, being activated by an increase in AMP (adenylate kinase converts 2 ADP into an ATP and AMP when ADP levels rise) [27]. AMPK inhibits ATP consuming processes (e.g. biosynthetic pathways) and turns on ATP-generating fatty acid oxidation and glycolysis via various routes, activating Acetyl CoA carboxylase and phosphofructokinase and recruiting the glucose transporter GLUT-4 to the plasma membrane [28].

### Calcium as a Master Regulator of Contraction and Energetics

Calcium acts as a master regulator: increased cytosolic calcium is the signal to initiate contraction (using ATP) but it also enters mitochondria via the mitochondrial calcium transporter, stimulating an increase in Krebs cycle reactions (several of the enzymes are calcium dependent), increasing mitochondrial ATP production in a fashion that matches the increased ATP requirement [29]. Mitochondrial calcium regulates pyruvate dehydrogenase, isocitrate dehydrogenase and alpha-ketoglutarate dehydrogenase [30], key steps in glucose metabolism, as well as the F1F0 ATPase responsible for ATP generation [31]. Via calcium-calmodulin-dependent kinase, calcium signals translocation of glucose and fat transporters from the sarcolemma to the cell membrane [32].

When beta-adrenoreceptor stimulation occurs, the redox state of NADH/NAD + and FADH2/FAD transiently oxidises (ADP accumulation accelerating respiration and using up reducing intermediates), followed by mitochondrial Ca2 + uptake which increases Krebs activity and regenerates redox states [33, 34]. If the mitochondrial calcium transporter is blocked, there is net oxidation of NADH/NAD + and FADH2/FAD reduced ETC activity [35], while if mitochondrial calcium transporter is deleted, pyruvate dehydrogenase activity is impaired causing increased fatty acid oxidation with a reduction in myocardial glycose oxidation [36] and there is delayed mitochondrial and contractile response to beta-adrenoreceptor stimulation [37]. Calcium is effluxed from mitochondria by the Na + -Ca2 + -Li + exchanger (NCLX), exchanged for sodium ions [38].

### Mechanotransduction

Mechanotransduction is the ability of cells to transduce physical stimuli into molecular signals. Biochemical and biophysical signalling pathways exist: biophysical pathways link the internal and external cellular dimensions together, leading to the exposure of activation domains on intracellular proteins when force is exerted while biochemical pathways are stress sensors (e.g. GTPases) activated when the cell is physically stressed [39]. The sarcomere, the intercalated disc and the sarcolemma are key sites of mechanotransduction [40].

Mechanical stretch results in calcium influx being induced by stretch-activated channels [39], with the initial increase in calcium spark being found to be mediated by components of the cytoskeleton [41].

## Adaptation to High Demand

### Calcium/Calmodulin-Dependent Protein Kinase

**Fig. 3:**
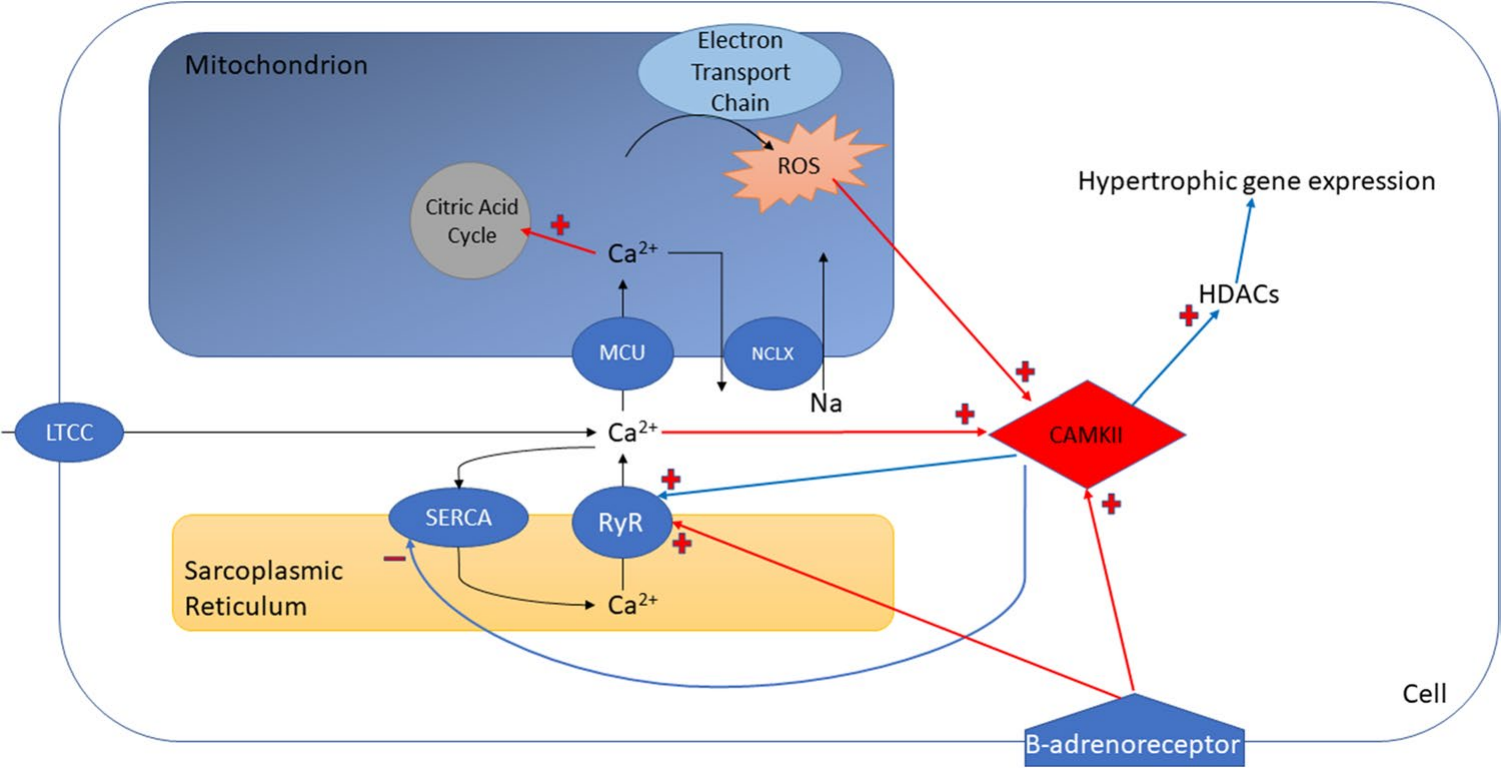
Signalling pathways linking calcium, reactive oxygen species (ROS), calcium-calmodulin-dependent kinase (CaMKII) and their downstream effects. Intracellular calcium enters the cell via L-type calcium channels (LTCC), enters the mitochondrion via mitochondrial calcium uniporter (MCU) to stimulate the citric acid cycle, activates CaMKII and is taken up into the sarcoplasmic reticulum via the sarcoplasm-endoplasmic reticulum ATPase (SERCA). CaMKII is activated by intracellular calcium, reactive oxygen species (ROS) production and beta-adrenoreceptors. It increases intracellular calcium levels by inhibiting SERCA and activating ryanodine receptor (RyR), as well as causing hypertrophic gene expression by activating histone deacetylases (HDACs). The sodium-calcium exchanger (NCLX) exchanges sodium and calcium across the mitochondrial membrane

Ca2 + /calmodulin-dependent protein kinase (CaMKII) is present in cardiac myocytes and activated by binding calcium/calmodulin complexes. Its activities are summarised in Fig. 3. It is involved in regulation of the excitation–contraction coupling, transducing the effect of beta-adrenoreceptor activation on the sinoatrial node to increase heart rate, acting as a physiological adaptor to higher heart rates by increasing strength of contraction and rate of relaxation in the short term and adjusting cardiac gene expression to cause hypertrophy in the long term [42]. However, oxidation by reactive oxygen species causes persistent activation (until reversed by the action of methionine sulfoxide reductase [43]) and over-activation causes structural and electric remodelling, leading to reduced contractility, arrhythmia and apoptosis [44].

CaMKII causes ‘facilitation’, the process by which inward currents though L-type calcium channels (LTCCs) are augmented as intracellular calcium concentrations rise [45], facilitating calcium entry into the cytoplasm with longer channel openings. CaMKII phosphorylates ryanodine receptors (RyR2, altering the channel open probability) and phospholamban (PLB, regulating calcium uptake) [46]: the sum of these actions being to increase contractile force during a shorter cardiac cycle. And, via entry into mitochondria, a concomitant increase in ATP production. CaMKII also increases re-uptake and sequestration of Ca2 + in the sarcoplasmic reticulum by SERCA activation, speeding diastolic relaxation [47].

## Impaired EC Coupling and Altered Calcium Handling Impair the Link Between Contraction and Mitochondrial Activity

Deterioration of cytosolic calcium handling weakens the link between contraction and ATP production. Sarcoplasmic reticulum calcium content is reduced (thanks to RyR2 leak) and SERCA activity is reduced. Systolic function is impaired as the magnitude and speed of the cytosolic Ca2 + transient is reduced, while diastolic function suffers owing to slowed [Ca] decay and higher diastolic [Ca].

The mitochondria and sarcoplasmic reticulum are closely coupled in [Ca2 +] [48]; hence, mitochondrial calcium content is reduced and the NCX operates in reverse mode, although this is insufficient to maintain [Ca2 +] [35]. A reduction in mitochondrial Ca2 + uptake results in relative oxidation of NADH/NAD + and FADH2/FAD + [35], giving rise to oxidative stress and ROS production, while correcting this defect in myocytes from failing hearts restored energy supply–demand matching [49]. Blocking L-type calcium channels or buffering calcium prevented stretch-induced apoptosis [50].

Other theories suggest that heart failure causes an increase in mitochondrial [Ca2 +]. It is worth noting that there are multiple populations of mitochondria within myocytes and effects may differ [51]. Mitochondrial calcium overload activates mitochondrial permeability transition pore (MPTP) opening, dissipating the gradient across the mitochondrial membrane and releasing pro-apoptotic factors leading to cell death (or, alternatively, if a large number of mitochondria are permeabilised the ATP deficit leads to cell necrosis) [52].

### Calcium/Calmodulin-Dependent Protein Kinase in Heart Failure

Unfortunately, CaMKII is a causative pathway in adverse remodelling, owing to its longer term effects elicited by changes in calcium homeostasis and altered calcium handling. Beta-adrenoreceptors associate with CaMKII to couple increased cAMP to CaMKII activation [53], while sustained beta-1 adrenoreceptor activation leads to increased free calcium and sarcoplasmic reticulum calcium overload culminating in CaMKII-mediated apoptosis [54]. In heart failure states, there is increased calcium influx, SR calcium leak and generally calcium overload, setting up a feed-forward effect where CaMKII further increases the activity of the LTCC, worsening calcium loading [45]. ROS is known to be increased in failing hearts and the activation of CaMKII by ROS links this to early after depolarisations via LTCC enhancement and impaired sodium channel activation, both of which are facilitated by CaMKII activation [55].

CaMKII appears to be a key link in translating pressure overload to failure as CaMKII knock-out mice subjected to aortic banding exhibited none of the features of failure (chamber dilation, ventricular dysfunction, fibrosis) that wild-type animals did but, interestingly, comparable hypertrophy [56]. CaMKII regulates HDACs which control hypertrophic gene pathways, for instance phosphorylating HDAC4 which results in activation of the transcription factor Myocyte Enhancer Factor 2 [57].

## The Failing Heart Is an Engine Out of Fuel

### Energetic Deficit

A unifying common phenomenon in the progression to failure is an energetic defect in the myocardium [58], an umbrella term describing changes in the Gibbs free energy of hydrolysis of ATP, reduction of the pool of phosphocreatine (the myocyte’s immediate temporal and spatial energy reserve) and impaired maximal ATP output. In humans, both the ratio of phosphocreatine to ATP [59] and the rate of phosphotransfer of ATP via creatine kinase [60] correlate with heart failure outcomes.

Whether this is a driver of heart failure or an epiphenomenon (or both) has yet to be conclusively proven, as while over-expression of creatine kinase attenuates dysfunction and improves survival in pressure overload–induced heart failure in mice [61], deletion does not induce heart failure progression [62]. Improving substrate availability with lipid supplementation does however improve PCr/ATP ratio in heart failure [63].

### Mitochondrial Morphology in Heart Failure

Morphological changes in heart failure include reduced mitochondrial area with mitochondrial fragmentation, cristae destruction, vacuolation and swelling [64], myelinization and membrane disruption [65]. Volume loading resulted in mitochondrial swelling and loss of cristae electron density, something that was preserved by administration of allopurinol or MitoQ, suggesting a role of ROS [66].

Mitochondrial fission is increased in heart failure and CaMKII phosphorylates and activates a protein involved in fission, dynamin-related protein 1 (Drp1) [67] and fusion is reduced due to reductions in fusion proteins such as optic atrophy 1 (OPA1) [68]. Mitophagy, the process by which damaged mitochondria are scavenged so that their contents can be re-used which is important for normal functioning [69], is impaired in heart failure with proteins required for the pathway reduced [70].

## Mitochondrial Changes Under Stress Driving Cellular Damage

### Reactive Oxygen Species

ROS is a broad term used to refer to reactive chemical species derived from oxygen, including OH^−^, O_2_^2−^ and H_2_O_2_. ‘Oxidative stress’ has come to be defined as the imbalance between ROS generation and antioxidant defence [71].

**Fig. 4:**
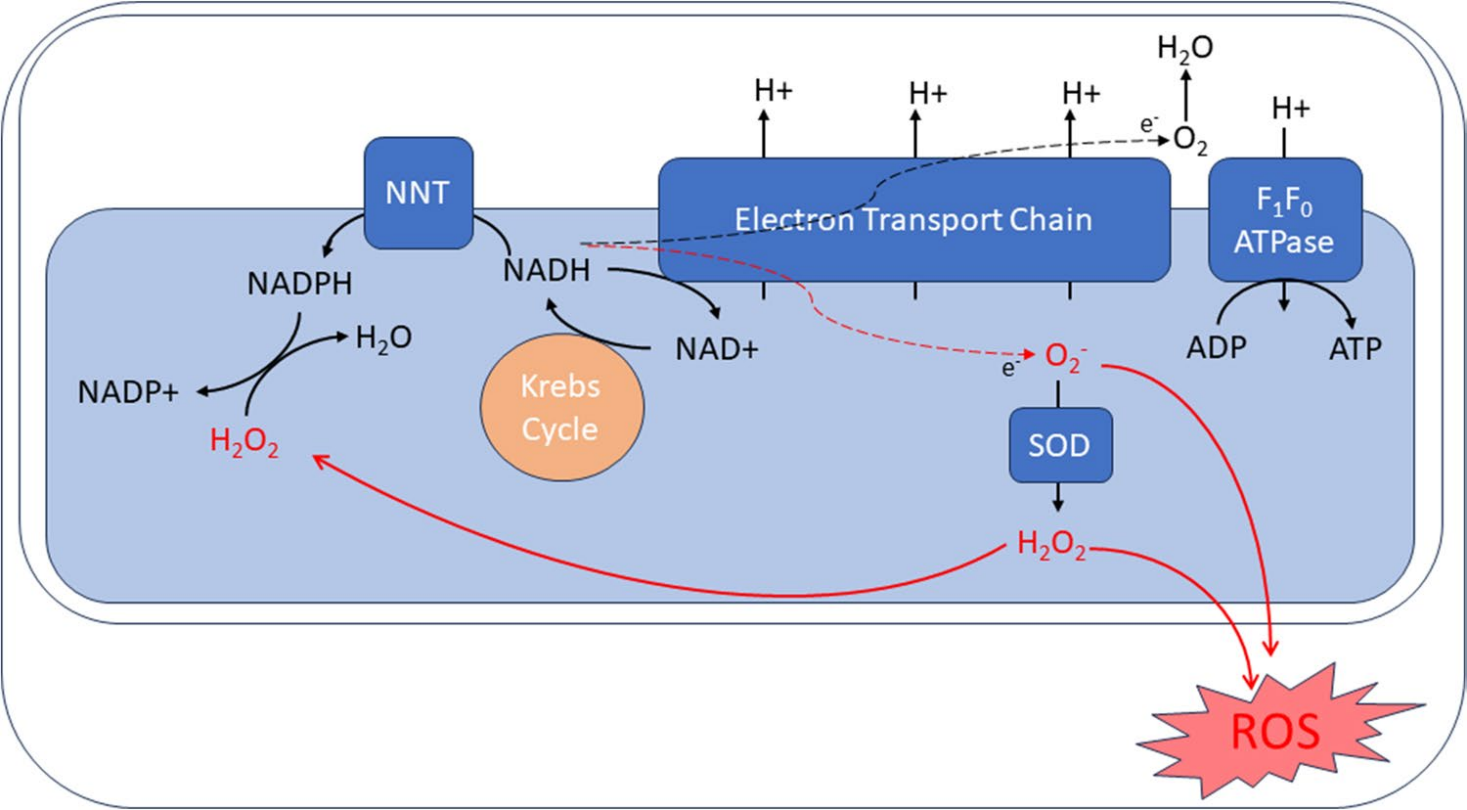
Pathways in reactive oxygen species (ROS) production. Electrons ‘leaking’ across the electron transport chain produce superoxide (O_2_^−^), which is converted to hydrogen peroxide (H_2_O_2_) by superoxide dysmutase (SOD), both of which are reactive oxygen species. Hydrogen peroxide may be converted to water by using NADPH as an electron donor. NADPH is formed from NADH via nicotinamide nucleotide transhydrogenase (NNT), so a reduction in NADH availability due to increased ATP demand will reduce antioxidant capacity within the cell

Electrons are normally transferred along the electron transport chain to reduce oxygen at complex IV and form water. Electrons ‘leaking’ (being transferred to other species prior to this) through complexes I and III in the electron transport chain result in oxygen being partially reduced to superoxide, which is converted to hydrogen peroxide by superoxide dismutase (SOD). Hydrogen peroxide is eliminated by antioxidant enzymes, mostly requiring NADPH as an electron donor. As mentioned above, impaired mitochondrial calcium uptake under pathological workloads results in ADP concentration outstripping ETC capacity, resulting in a less oxidised NADH/NAD + pair: this reduces the NADH available to form the antioxidant NADPH [72]. Succinate accumulates during cardiac ischaemia and induces reverse electron transport during reperfusion, causing a damaging surge of ROS generation [73], proposed as the mechanism for ischaemia–reperfusion injury. Deterioration of mitochondrial calcium uptake seen in heart failure results in a fall in regeneration of the reduced forms of NADH and NADPH, causing energetic deficits and oxidative stress. This pathway is summarised in Fig. 4.

ROS accumulation has been reported in cardiomyopathies and heart failure, although the direct cellular mechanisms leading to its production have not been fully elucidated: there may be multiple mechanisms and they may vary between types of heart failure. Mechanical stretch appears to directly activate ROS production via the Rac1-ROS pathway [74] and direct stretch on heart cells causes activation of NOX2 leading to ROS generation in a mechanochemical manner which is microtubule dependent, referred to as X-ROS signalling [75]. X-ROS signalling is responsible for experimentally inducing arrhythmogenic calcium waves in Duchenne muscular dystrophy cells [76] and high levels of mechanical stress cause SERCA inactivation via peroxynitrite-mediated nitration [77], leading to calcium load.

As well as in CaMKII activation, ROS activate hypertrophy signalling kinases [78], cause damage to mitochondrial [79] and cellular DNA [80], cause apoptosis [81], activate secretion of matrix metalloproteases [80] and increase open possibility of ryanodine receptors, thus producing arrhythmia [82]. ROS can also beget further ROS production in a vicious cycle [83].

However, ROS has regulatory functions in other tissues and may also have some in the heart [51] so it should not be considered to be solely damaging. Nonetheless, mitochondrial ROS are causally related to HF progression in a variety of animal models [72].

### Mitochondrial Permeability Transition Pore

The mitochondrial permeability transition pore (MPTP) is a key mechanism for mediating cardiac dysfunction and cell death: when it opens, it allows passages of solutes up to 1.5 kDa in size [84], resulting in inner membrane potential collapse, respiratory chain uncoupling and hence halting mitochrondrial ATP synthesis. Eventually, mitochondrial swelling, rupture and necrotic cell death occur [85]. It is primarily triggered by calcium levels within the mitochondrial matrix; however, oxidative stress, adenine nucleotide depletion, elevated phosphate and pH/mitochondrial depolarisation will also open it, and other divalent cations (e.g. magnesium) will inhibit its opening [86]. Despite much work, which protein (or proteins) make up the MPTP is still uncertain, with cyclophilin-D, adenine nucleotide translocase, mitochondrial phosphate carrier, F_1_F_0_ ATP synthase and the voltage-dependent anion channel all proposed [86, 87].

The MPTP has some physiological functions and is involved in ROS signalling (producing ‘flashes’ of superoxide [88]), cardiomyocyte development [89] and mitochondrial calcium efflux. Transient openings of the MPTP have been proposed as a mechanism to reduce calcium concentrations within the mitochondrion when in sustained calcium overload [90].

It is easy to see how ischaemia will lead to MPTP opening, as ATP levels dissipate, ROS are produced and calcium levels climb [91]. In addition, the MPTP remains open in reperfusion and is mechanistic in ischaemia–reperfusion injury [92], while playing a role in ischaemic preconditioning [93].

As calcium dysregulation, ATP depletion and ROS production are all present in heart failure, it has been hypothesised that MPTP is involved in HF progression [87]. CypD-deficient mice were protected against cardiomyopathy induced by calcium overload [94], but display increased hypertrophy and mortality in response to pressure overload [95], perhaps illustrating some of the protective functions of MPTP.

### Senescence

Senescence is usually considered a state of cell cycle arrest; however, as cardiomyocytes are terminally differentiated, they must be considered differently [96]. Senescent cardiomyocytes exhibit functional alterations (dysregulated contraction, irregular shortening, elevated pacing frequency), structural alterations (enlarged size, mitochondrial dysfunction, shortened telomeres) and production of factors affecting neighbouring cells (SASP: senescence-associated secreting phenotype) [97].

Mitochondrial dysfunction occurs in senescence, in response to p53 activation [69] and downstream signalling from hypoxia [98].

ROS are implicated in the pathophysiology of senescence, causing DNA damage. Telomere shortening being the commonest feature, which has been shown to drive a senescent-type phenotype [99], while DNA damage links to NAD + depletion and consequent energy loss via PARP1 [100].

## Areas for Future Work

### Mitochondrial Damage-Associated Molecular Patterns

Damaged tissue releases DAMPs (damage-associated molecular patterns), which act on immune cells but also a wide range of tissues including cardiomyocytes, cardiac fibroblasts and cardiac endothelial cells. Mitochondrial DAMPs (mDAMPs) are those released from within mitochondria, including mitochondrial DNA, N-formyl peptides, cardiolipin, mitochondrial transcription factor A (TFAM), ATP, reactive oxygen species (ROS), succinate and cytochrome C [101]. These may be released by damaged or stressed mitochondria into the cytosol, or from necrotic cells into the extracellular space. Cytochrome C, mitochondrial DNA and succinate have been shown to correlate with markers of myocardial injury such as troponin or myocardial oedema [101] and activate healthy PBMCs [102]. Mitochondrial DNA [103] and ER stress [104] activate the cGAS-STING pathway, which is involved in myocyte senescence [105], as well as activating NF-kB and IRF-3 pathways, leading to TGF-B1 production and fibrosis [106].

Mitochondrial DNA escaping autophagy has been shown to cause TLR9-mediated inflammatory responses resulting in infiltration by inflammatory cells and inflammatory cytokine expression leading to inflammation and cardiac chamber dilation in mice [107].

## Conclusions

**Fig. 5:**
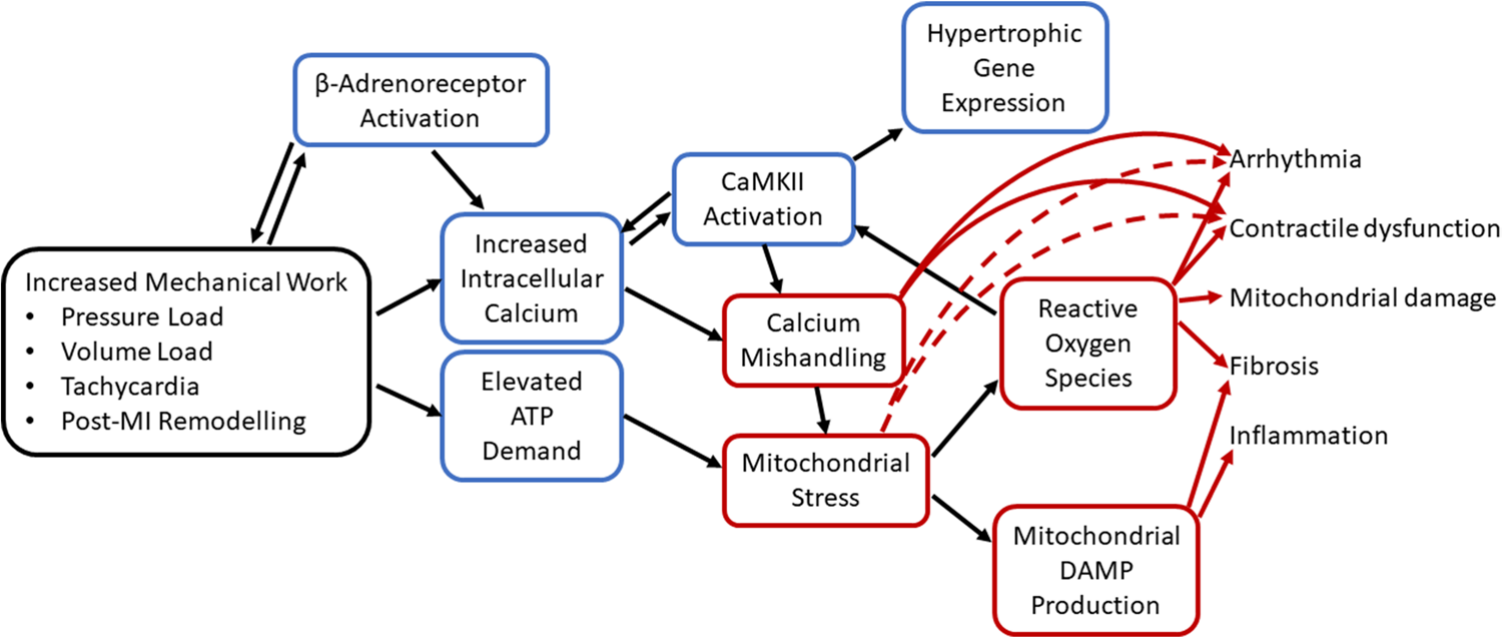
Overall schematic linking increased mechanical work to damaging processes causing heart failure progression. Blue boxes indicate adaptive processes and red boxes maladaptive processes

Sustained high cardiac workload sets up a vicious cycle of mitochondrial stress and calcium mishandling. Reduced calcium entry into mitochondria worsens the mismatch between energy supply and demand and oxidative stress results in production of reactive oxygen species, causing CaMKII activation and further deleterious changes to calcium handling. Beta-adrenoreceptor activation feeds directly into calcium mishandling and indirectly via CaMKII activation. Production of ROS and mitochondrial DAMPs causes cellular and tissue level damage. These processes link increased cardiac workload to the progression of heart failure (Fig. 5).

## Data Availability

Not applicable.
